# COVID-19 Infection Risk and Depressive Symptoms Among Young Adults During Quarantine: The Moderating Role of Grit and Social Support

**DOI:** 10.3389/fpsyg.2020.577942

**Published:** 2021-01-08

**Authors:** Jie Hou, Qingyun Yu, Xiaoyu Lan

**Affiliations:** ^1^College of General Aviation, Jingchu University of Technology, Jingmen, China; ^2^Counseling Center for Mental Health Education, Jingchu University of Technology, Jingmen, China; ^3^Department of Developmental Psychology and Socialization, University of Padua, Padua, Italy

**Keywords:** depressive symptoms, coronavirus disease 2019 infection risk, grit, social support, young adults

## Abstract

Prior research has demonstrated that the adverse consequences of the coronavirus disease 2019 (COVID-19) pandemic may go beyond its economic hardships and physical health concerns, having a significant influence on psychological distress for individuals under quarantine. Nevertheless, relatively little attention has been paid to exploring the risk and protective factors in the link between COVID-19 infection risk and psychological distress among young adults. Following a socioecological framework, the current study examines the moderating role of grit (perseverance and consistency) and social support in the association between COVID-19 infection risk and depressive symptoms. A sample of 1,251 young adults under home quarantine (62.6% female; *M*_*age*_ = 20.92 years, *SD* = 1.47; age ranged from 18 to 25 years) was involved in this study, and they were asked to complete a set of self-reported questionnaires online. Results of a linear regression analysis exhibited that COVID-19 infection risk was positively associated with depressive symptoms in young adults in quarantine. Moreover, moderation analyses showed that this association was moderated by perseverance and social support. To be specific, for those reporting higher levels of social support, this linkage was not significantly positive; in contrast, for those reporting lower levels of social support, perseverance was a significant protective factor for depressive symptoms when young adults were exposed to a high infection risk of COVID-19. The current study suggests that greater social support is essential to helping young adults deal with possible negative emotions in the context of the COVID-19 pandemic. Moreover, university-based counseling services should pay specific attention to those young adults with relatively insufficient social support resources and low levels of perseverance.

## Introduction

The outbreak and spread of the coronavirus [hereafter referred to as “COVID-19” (coronavirus disease 2019)] has become a global public health concern (Altena et al., 2020; Tian et al., 2020; Xiang et al., 2020). To control the transmission of COVID-19, many countries have implemented strict quarantine policies (Brooks et al., 2020; Webster et al., 2020). Although quarantines have been demonstrated to limit exposure to and the spread of contagious diseases, there are potential concerns with quarantine practices, as isolation can lead to psychological distress, including the emergence of depressive symptoms (Liu et al., 2020; Huang and Zhao, 2020; Meng et al., 2020; Ransing et al., 2020). Despite a burgeoning body of empirical literature documenting the variables correlated with depressive symptoms in COVID-19 quarantine, quarantine impact as a function of age has received little research attention.

Young adults encounter significant life changes and challenges due to developmental shifts, including commitment to intimate relationships, job seeking, pursuing higher education, and taking on new social responsibilities (Arnett, 2000; Lan et al., 2019a). For young adults who are quarantined at home with their parents or relatives, the stress caused by such a sharp change in their environment (as they shift from sudden personal independence back to restricted mobility and limited socialization options) may be pronounced, thereby resulting in higher vulnerability to psychological distress (Riggs and Han, 2009). Quarantine and physical distancing practices also generate widespread social media use to maintain regular communications with significant others (Parola et al., 2020; Xin et al., 2020); this is particularly evident in young adults, who represent a sizable majority of social media users (China Internet Network Information Center [CINIC], 2019). Although mass social media exposure and social networking site involvement may facilitate communication between individuals and soothe individuals’ psychological distress, it also increases exposure to “panic information” online. Indeed, in the initial stage of the COVID-19 pandemic, misleading rumors, corona-phobia, and conspiracy theories about its origin circulated around the globe, influencing individuals’ well-being and their reactions to the COVID-19 pandemic in negative ways (Depoux et al., 2020).

Research suggests that depressive symptoms during young adulthood may reduce one’s ability to deal with major developmental tasks, which in turn leads to negative consequences in later life, such as low achievement, occupational difficulties, and social dysfunction (Schulenberg and Zarrett, 2006; Salmela-Aro et al., 2008). Considering that intervention or prevention during this transitional period can positively influence the direction of development (Masten, 2001; Galambos et al., 2006), researchers need to investigate the risk and protective factors for depressive symptoms in young adults in quarantine.

To examine the risk and protective factors for depressive symptoms, we refer to the socioecological framework (Bronfenbrenner, 2001). This framework has emerged as one of the meta-paradigms for understanding the multiple correlates related to depressive symptoms (Buzi et al., 2015). According to this framework, an individual’s depressive symptoms occur within the socioecological context in which that individual exists and unfold within dynamic person–context interactions, influenced by such factors as individual characteristics (e.g., grit) and the features of the social environment (e.g., the presence of social support). Given this complexity, it is necessary to ascertain how multiple factors impact young adults’ depressive symptoms in response to COVID-19 infection risk, independently or in interactive ways, rather than examining each factor solely in isolation (Zhou et al., 2012; Lan and Zhang, 2019). Such a framework has been applied to assess the correlates of depressive symptoms in numerous well-established empirical studies, with fruitful outcomes (e.g., Buzi et al., 2015; Lan and Wang, 2020b).

To summarize, the current study investigates the association between COVID-19 infection risk and depressive symptoms among young Chinese adults in quarantine; specific attention is paid to the possible risk and protective factors related to grit (perseverance and passion for long-term goals) and social support within this population. In the following section, we present a literature review of study variables, starting from the presentation of the link between COVID-19 infection risk and depressive symptoms.

### Coronavirus Disease 2019 Infection Risk and Depressive Symptoms

Infection risk is defined as the potential to acquire a disease that represents a physical threat or danger to one’s health, particularly for those with suspected symptoms or who have close contact with infected/suspected individuals (Lau et al., 2008; Chen et al., 2020; Simione and Gnagnarella, 2020). Due to substantial scientific uncertainties about disease properties with the outbreak of a new virus, possible infection risk may trigger widespread public concerns that then translate into psychological distress (Smith, 2006; Lau et al., 2008). Moreover, the relationship between infection risk and depressive symptoms can be further heightened in quarantine, as disruptions to social support networks, significant economic hardships, fears of physical safety, and the experience of illness and death among family and friends can bring additional stress for young adults, leading to negative psychological outcomes (Brooks et al., 2020; Liu et al., 2020; Ransing et al., 2020).

A burgeoning body of research has documented the effects of the COVID-19 pandemic on adults’ psychological outcomes in quarantine; for example, Ammerman et al. (2020) have revealed that COVID-19-related experiences are positively associated with suicidal thoughts and behaviors in adults. Moreover, Nguyen et al. (2020) have reported that adults with suspected COVID-19 symptoms exhibit a higher depression likelihood and lower health-related quality of life than those without suspected symptoms. In terms of Chinese adults in quarantine, research has documented a high prevalence of mental health problems, including depression and anxiety (Gao et al., 2020; Hu et al., 2020). Despite these relevant findings, little research attention has been paid to young adults in quarantine, and the possible risk and protective factors in the association between COVID-19 infection risk and depressive symptoms are less explored. Attempting to address these research gaps, we examine the moderating role of two dimensions of grit in the relationship between infection risk and depressive symptoms in Chinese young adults in quarantine.

### Grit

Grit is conceptualized as passion and perseverance in the pursuit of long-term goals (Duckworth et al., 2007; Duckworth, 2016). In recent years, many empirical studies have demonstrated the beneficial effects of grit on various psychological functions in individuals (see a meta-analysis by Credé et al., 2017), including young Chinese adults (Zhang et al., 2018; Jin et al., 2019). Concerning the link between grit and depressive symptoms, Datu et al. (2019) have found that it is negatively associated with depressive symptoms in their investigation of the presence of meaning in life among high school students. The authors explain that “gritty” individuals may consider possible life challenges as an inevitable part of goal-striving and interpret them more positively than those with lower levels of grit. Thus, such individuals are less prone to depressive episodes; this finding was also replicated in a sample of university students (Musumari et al., 2018). In terms of Chinese young adults, Lan et al. (2019b) have demonstrated that grit is negatively linked to depressive symptoms for young adults who report early traumatic experiences and that this effect is heightened in the context of high levels of peer support. Although informative, a noted limitation of the existing literature is that much of the research has focused on overall grit scores, neglecting the possible distinctions between different grit facets on young adults’ depressive symptoms (Luo et al., 2020).

Duckworth et al. (2007) have proposed that grit contains two distinct facets: perseverance of effort (hereafter, “perseverance”—sustaining personal effort and determination to accomplish a goal) and consistency of interests (hereafter, “consistency”—commitment to interests that may lead to goal achievement). An emerging body of research has noted that these two facets may reflect independent constructs that contribute to individuals’ adaptive functions in distinctive ways (Datu et al., 2016; Lan, 2020; Lan and Radin, 2020; Luo et al., 2020); for example, research has shown that the buffering role of perseverance on externalizing problem behavior is more salient than consistency, especially for those who confront personal vulnerabilities (Lan and Radin, 2020). The authors explain these findings in terms of Chinese cultural values, in which perseverance and diligence are highly valued, while consistency is not. Despite this finding, it is still unclear whether perseverance and consistency are linked to internalizing problems, such as depressive symptoms, in a distinctive manner.

Moreover, according to the socioecological framework (Bronfenbrenner, 2001), a favorable social context may magnify the buffering effect of individual characteristics (e.g., grit) on depressive symptoms in young adults. For example, Clark et al. (2020) found that the positive association between grit and academic achievement is stronger for high school students reporting high social support from teachers, explaining that when students perceive greater social support, they are more likely to study toward long-term goals with passion and achieve higher grades. Although this empirical finding gives us some possible indications, it is still unclear how those variables may contribute in interactive ways to the association between COVID-19 infection risk and depressive symptoms, as a study of high school students and academic achievement differs substantially from the context of young adults and their emotional reactions to quarantine. Given the above findings, however, we attempt to investigate the moderating role of social support in the association between COVID-19 infection risk and depressive symptoms in young adults in addition to the roles of the two dimensions of grit.

### Social Support

Perceived social support is defined as an individual’s subjective feelings about and evaluations of support offered by other individuals in the external environment (Zimet et al., 1988). Extant literature has demonstrated that social support is paramount to adaptive psychological functioning in young adults (see meta-analyses by Rueger et al., 2016; Lei et al., 2018). This perspective is assumed to be more pronounced in Chinese society, which emphasizes group harmony and appropriate interpersonal interactions (Bond, 2010; Chen, 2010). In addition to the essential role of family relations in individuals’ social support systems, transforming individuals outside the family system into kin by a reciprocal exchange of favors is also highly underscored in the context of Chinese culture (Bond, 2010; Lan, 2020).

Although studies have demonstrated that social support is an essential protective factor for depressive symptoms (Rueger et al., 2016; Lei et al., 2018), it is still unclear under which conditions perceived social support works to this end. In particular, the buffering effect model suggests that the positive impact of social support in reducing psychological distress only occurs when under stress (Auerbach et al., 2011; Dumitrache et al., 2017; Wang et al., 2020). Indeed, when individuals encounter obstacles or experience negative emotions in their daily lives, they are more likely to ask significant others for support and comfort (Vélez et al., 2016); for example, Dumitrache et al. (2017) discovered that social support buffers against the impact of negative perceived health status and life satisfaction in adults. More precisely, when exhibiting a negative evaluation of health status, social support can significantly improve adults’ life satisfaction. This finding suggests that, when reporting high COVID-19 infection risk, social support may help young adults to avoid generating negative emotional states, such as depressive symptoms.

### The Current Study

Given the possible vulnerability of young adults under COVID-19 quarantine to depressive symptoms, we aim to explore several risk and protective factors, illustrating individual differences in depressive symptom profiles. Specifically, we conducted a simultaneous investigation of the moderating role of two dimensions of grit and social support in the relationship between infection risk, quarantine, and depressive symptoms. In accordance with the literature reviewed above, we examine the following hypotheses:

Hypothesis 1: COVID-19 infection risk is positively associated with depressive symptoms in young adults under quarantine.

Hypothesis 2: When reporting high levels of infection risk, young adults with higher levels of grit and/or social support may report lower levels of depressive symptoms than those exhibiting lower levels of grit and/or social support. Moreover, considering the possible distinctions between the two facets of grit, we expect that the moderating role of perseverance will be more salient than consistency in the association mentioned above.

Furthermore, prior research has indicated that sociodemographic variables are potentially linked to depressive symptoms in young adults (Galambos et al., 2006; Pettit et al., 2011; Cao et al., 2020). Additionally, fear of infection, rather than actual infection risk, may increase adults’ psychological distress (Brooks et al., 2020), while the number of close family members during quarantine may influence the quality of social support (Chou and Chi, 2001), potentially blurring understudied associations; social media is also associated with mental health problems for adults during the COVID-19 pandemic (Gao et al., 2020). Taking these findings together, we consider age, gender, family socioeconomic status (SES), fear of infection, the number of close family members present during quarantine (hereafter, “number”), and the time of social media exposure to COVID-19-related information (hereafter, “time”) as confounding variables in this study.

## Methods

### Participants and Procedures

The current study is based on a larger research project concerning the psychosocial adjustment of young adults during COVID-19 quarantine^1^. Apart from the variables employed in this study, we also included other constructs (e.g., prosocial behavior, subjective well-being, and mindfulness). Before data collection, the current study was ethically approved by the institutional review board affiliated with the Jingchu University of Technology. Based on a convenience sampling method, we invited college students in Hubei Province (Central China; the province with the largest number of confirmed cases) to participate in this study on a voluntary basis. Prior to the presentation of targeted questionnaires, informed consent and a brief statement of participants’ rights were presented. Only on the condition that participants approved this piece of information would the follow-up survey start. In the meantime, confidentiality and anonymity of this investigation were strictly guaranteed through all research processes. Due to the limited research funding, participants did not receive a monetary reward after their participation. However, participants who took part in this investigation would get free access to university-based counseling services. We reasoned that this non-monetary incentive would potentially enhance the participation rate.

Data collection was administered online (Wenjuanwang) in March 2020. At this moment, young adults were strictly quarantined at home for almost 2 months due to COVID-19^2^. In the current study, we ordered the questionnaires based on the following considerations: (a) we put possible independent variables at the beginning and dependent variables at the end of this investigation; and (b) we prioritized positive constructs at the end of this investigation to avoid possible “spillover” effects, as negative constructs at the end might influence young adults’ mood and their subsequent activities. Concerning study variables in the current study, the questionnaires were administered in the following order: COVID-19 infection risk, depressive symptoms, grit, and social support. As recommended by the procedures of conducting Web-based data collection (Lan et al., 2019b), missing values and skipping the questions would prompt a reminder to double-check before submission. This was done to avoid missing out on any items. Still, participants were free to skip any specific question or withdraw from the research process, as we highlighted in the brief statement at the beginning of this survey.

The original sample size consisted of 2,100 adults, and approximately 800 adults were excluded from the current study based on the following considerations. First, to ensure valid and attentive responding, we omitted the participants who failed to pass the time completion check. The mean completion time of this investigation was 1,209 s (approximately 20 min), and participants whose completion time was below 3 standard deviations of the mean completion time were eliminated from this study. Second, we deleted the participants whose age range exceeded our research interest (i.e., 18–25 years). The rationale for this age range is congruent with Arnett’s developmental theory of young adulthood (Arnett, 2000; Lan and Wang, 2020a).

Finally, a total sample of 1,251 young adults (62.6% female) was involved in the current study. The mean age of participants was 20.92 years (*SD* = 1.47, range = 18–25). The majority of the sample was identified as the Han ethnic group (the majority ethnic group in China; 91.5%, *n* = 1,145). Moreover, 569 (45.5%) of these participants were in their second year of college; 559 (44.7%) were in their third year; and 123 (9.8%) were in their fourth year. Concerning the educational background of their parents, 24.5% and 39.0% of fathers and mothers have completed primary school education or lower, 46.5% and 40.4% have completed middle school education, 20.5% and 16.1% have completed high school education, and 8.5% and 4.4% have completed undergraduate education or higher. Concerning family income, most young adults reported that their monthly family incomes were 500–1,000 US dollars.

### Measures

#### Depressive Symptoms

Depressive symptoms were measured by the Center for Epidemiological Studies-Depression (CES-D) scale (Radloff, 1977), which is regarded as one of the widely used assessment tools for adults’ depressive symptoms (Demirkan et al., 2016). Participants were asked to rate this 20-item scale, measuring the frequency with which the symptoms occurred in the last week, ranging from 0 (rarely) to 3 (sometimes). One of the item examples is, “I was bothered by things that usually do not bother me.” All these items were summarized to yield a total score, with higher scores indicating severe depressive symptoms. Prior research has exhibited good internal consistency of the CES-D in Chinese young adults (Lan et al., 2019b). In this study, Cronbach’s alpha was 0.88.

#### Coronavirus Disease 2019 Infection Risk

We utilized four face-valid items to evaluate the possibility of COVID-19 infection risk. These items were modified based on the health code released by the National Health Commission of China [NHCC] (2020b). The health code (with distinct colors: green, yellow, or red) is automatically generated when individuals use the software (i.e., Alipay) on their smartphones to answer targeted questions. This code aims to evaluate individuals’ infection risk of COVID-19 and assess whether individuals should be allowed to go into public spaces. Due to the necessity of generating this health code during quarantine, most participants may already know how to establish it before completing this investigation. To be specific, in our research batteries, participants were asked to indicate whether they had experienced the following events in the past 2 weeks on a dichotomous scale (0 = no, 1 = yes): suspected symptoms, close contact with a probable or confirmed case, close contact with individuals from epidemic centers, and close contact with individuals from epidemic areas. Item responses were summed to yield a total score, with higher scores indicating higher levels of infection risk of COVID-19. We did not compute the internal reliability of these items, as these events did not necessarily occur together.

#### Grit

Grit was assessed by the brief self-report version of the Grit Scale (Duckworth and Quinn, 2009), which measures trait-level perseverance and passion for long-term goals. This scale consists of eight items and retains the two-factor structure of the original Grit Scale with four items for each dimension (i.e., perseverance and consistency). Item examples are, “setbacks do not discourage me (perseverance)”; “new ideas and projects sometimes distract me from previous ones (consistency).” Young adults were asked to rate each item on a Likert scale ranging from 1 (*not like me at all*) to 5 (*very much like me*). The average score of all these items (per dimension) was calculated, with a higher value indicating greater perseverance and consistency, respectively. Prior research has shown good internal consistency of this scale in Chinese young adults (Lan, 2019; Luo et al., 2020). In this study, Cronbach’s alpha was 0.82 and 0.83 for perseverance and consistency.

#### Social Support

Social support was measured by the 12-item Multidimensional Scale of Perceived Social Support (MSPSS; Zimet et al., 1988). The MSPSS assesses three sources of support: family, friends, and significant others. One of the item examples is, “I have friends with whom I can share my joys and sorrows (on the friends’ domain).” It is noteworthy that the current sample was a group of college students who were more likely to be engaged in romantic relationships. Therefore, significant others are most likely to be represented by their close friends in romantic relationships, which has been documented in prior research of Chinese college students (Zhao et al., 2014). This is different from previous study using a sample of Chinese adolescents (Chou, 2000), as friends and significant others mostly overlap for adolescent populations. Participants were asked to rate each item from 1 (*very strongly disagree*) to 7 (*very strongly agree*). All these items were averaged to obtain an overall score, with a higher score indicating a higher perception of social support. Although it would be informative to unpack the roles of different social support resources in college students, we retained the total score of social support instead of analyzing each dimension. This is because we are interested in understanding the moderating role of grit and social support in the association between COVID-19 infection risk and depressive symptoms. In this study, we had already unpacked the role of different dimensions of grit, and several interactions involved in linear regression would complicate the results’ interpretation. Moreover, the statistical power would be quite questionable in this context, as too many predictors were inserted inside one single linear regression. Prior research has demonstrated good internal consistency of the MSPSS in Chinese college students (Kong et al., 2012; Zhao et al., 2014). In this study, Cronbach’s alpha was 0.97.

#### Sociodemographic Characteristics

We asked young adults to indicate their sociodemographic characteristics, including age, gender, ethnicity, grade, educational level of their parents, and monthly family income. In terms of parental education, five categories were available: (1) primary school graduation or lower, (2) middle school graduation, (3) high school graduation, (4) bachelor’s degree graduation, and (5) master’s degree graduation or higher. Concerning monthly family income, several options were provided, ranging from (lower than 1,000 RMB) to (higher than 20,000 RMB). As suggested by prior research (Lan and Wang, 2020b), the scores of fathers’ educational background, mothers’ educational background, and monthly family income were first standardized and then summed into a composite score, with higher scores indicating higher levels of family SES.

#### Control Variables

Apart from the possible influences of sociodemographic variables (age, gender, and SES; see the “Sociodemographic Characteristics” section) on depressive symptoms of young adults in quarantine, we also controlled some COVID-19-related variables. First, we used eight modified items initially developed by Mertens et al. (2020) to assess the fear of infection to COVID-19. Participants were asked to rate each item on a 5-point Likert scale ranging from 1 (*strongly disagree*) *to* 5 (*strongly agree*). One of the item examples is, “I am worried that friends or family members will be infected.” Prior research has shown good internal consistency of this scale (Mertens et al., 2020). In this study, Cronbach’s alpha was 0.91. Second, we required young adults to indicate how many people stay together in quarantine, and a 6-point scale was administered, ranging from 1 (*alone*) to (*five people or more*). Third, we asked young adults to report how many hours per day they spent getting access to news and information related to COVID-19 in social media in the past 2 weeks. A 9-point scale, ranging from 1 (*never*) to 9 (*6 h or more*), was adopted for this item.

### Data Analytical Plan

Data analyses were conducted using IBM SPSS Statistics Version 21.0 (IBM Corp, 2012) and Jamovi 1.1.9.0 (The Jamovi Project, 2020). Original dataset of this study is publicly available on the Open Science Framework repository (^3^ doi: 10.17605/OSF.IO/8D6P9). We first performed descriptive statistics (means, standard deviations, and range of scores) and zero-order correlations among study variables to have an overview of study variables.

In terms of the research objective, we used multiple linear regression analysis to ascertain the associations of COVID-19 infection risk, two dimensions of grit, and social support with depressive symptoms, controlling for age, gender, family SES, fear of infection, the number of close family members present during quarantine, and the time of social media exposure to COVID-19-related information^4^. Multiple linear regression analysis refers to a statistical technique that uses several explanatory variables to predict a dependent variable (Nathans et al., 2012). Multiple linear regression analysis allows researchers to examine hypotheses involving moderation effects. Moderation effects are typically evaluated by testing the significance of a multiplicative term consisting of the product between two or more predictors controlling for associated lower order main effects (Aiken and West, 1991; Aiken et al., 2003). When a significant interaction is found, simple slope analysis is applied to probe the structure of the relation (Aiken and West, 1991; Preacher et al., 2006).

In line with our second hypothesis, we employed the scores of two dimensions of grit instead of the overall score of grit in linear regression analysis. This was done to address the limitations of prior research (see section “Introduction”) and further examine whether perseverance and consistency may distinctively moderate study associations. Although the cultural emphasis of perseverance in Chinese society has been documented, it is still essential to simultaneously incorporate perseverance and consistency, given the theoretical relevance of these two dimensions (Duckworth et al., 2007) and the scarcity of literature concerning the association between these two dimensions and internalizing problems. Apart from the main effects and confounding variables, a set of two- and three-way multiplicative interaction terms was added (infection risk × perseverance, infection risk × consistency, infection risk × social support, perseverance × social support, consistency × social support, infection risk × perseverance × social support, infection risk × consistency × social support) in the linear model. The inclusion of these interaction terms was to examine whether two dimensions of grit and social support might moderate the association between COVID-19 infection risk and depressive symptoms. It should be noted that creating the three-way interaction term requires researchers to establish low levels of interaction terms (i.e., two-way interaction) sequentially, although the interaction between two dimensions of grit and social support was not our research aim (see also Ma et al., 2020).

## Results

### Descriptive Statistics

Means and standard deviations for study variables are presented in Table 1. The mean scores of perseverance (*M* = 3.40; *SD* = 0.71; range from 1 to 7), consistency (*M* = 3.32; *SD* = 0.74; range from 1 to 7), and social support (*M* = 5.37; *SD* = 1.03; range from 1 to 7), as perceived by the participants, were generally high. However, the mean value of COVID-19 infection risk was only 0.23 (*SD* = 0.49), taking into account that the measure can take on values in the range of 0–4, and the mean value of depressive symptoms was only 12.51 (*SD* = 7.33) on a scale that the total score could range from 0 to 60.

**TABLE 1:** Descriptive statistics and bivariate correlations of study variables for Chinese young adults in quarantine.

	***M***	***SD***	**Range**	**1**	**2**	**3**	**4**	**5**	**6**	**7**	**8**	**9**	**10**	**11**
1. COVID-19 infection risk	0.23	0.49	0–4	−										
2. Perseverance	3.40	0.71	1–5	−0.02	−									
3. Consistency	3.32	0.74	1–5	^–^0.05*	0.13***	−								
4. Social Support	5.37	1.03	1–7	−0.01	0.44***	0.17***	−							
5. Depressive symptoms	12.51	7.33	0–60	0.07*	−0.11***	−0.22***	−0.16***	−						
6. Age	20.92	1.47	18–25	−0.04	0.09***	0.10***	0.06*	−0.01	−					
7. Male^*a*^	−	−	1–2	−0.01	−0.02	0.02	0.13***	0.02	0.01	−				
8. Socioeconomic status	6.88	2.12	3–15	0.08**	0.03	−0.01	0.10***	−0.03	−0.08**	−0.02	−			
9. Fear of infection	1.95	0.80	1–5	0.02	−0.03	−0.09***	−0.08**	0.18***	0.01	0.02	−0.14***	−		
10. Number	4.37	1.18	1–6	0.07*	0.01	0.02	−0.01	−0.02	−0.04	0.13***	−0.07*	0.06*	−	
11. Time	3.11	1.38	1–9	0.03	0.07*	−0.01	0.01	0.04	0.09***	0.02	−0.01	0.13***	0.02	−

N = 1,251. ^*a*^Gender was regarded as a dummy variable, and we specified male as a reference group in this study. M, mean; SD, standard deviation; SES, socioeconomic status. The standardized score of SES ranged from -4.13 to 8.62. Number refers to the number of close family members present during quarantine, and time reflects how many hours per day young adults spent getting access to news and information related to coronavirus disease 2019 (COVID-19) in social media. *p < 0.05, **p < 0.01, and ***p < 0.001.

Table 1 also presents bivariate correlations among study variables. COVID-19 infection risk (*r* = 0.07, *p* < 0.05) was positively related to depressive symptoms; perseverance (*r* = -0.11, *p* < 0.001), consistency (*r* = -0.22, *p* < 0.001), and social support (*r* = -0.16, *p* < 0.001) were each negatively associated with depressive symptoms. In terms of covariates, fear of infection (*r* = 0.18, *p* < 0.001) was positively linked to depressive symptoms, whereas other confounding variables (i.e., age, gender, family SES, number, and time) did not exhibit significant correlations with depressive symptoms.

### Associations of Coronavirus Disease 2019 Infection Risk, Grit, and Social Support With Depressive Symptoms

Given that there were some low-to-moderate correlations between the predictors, the multicollinearity issue may have an adverse influence on estimated coefficients in the linear regression (Mansfield and Helms, 1982). Therefore, before presenting the results of the linear regression analysis, we calculated the variance inflation factors (VIFs) of the predictors. The results exhibited that the range of VIFs of the predictors was from 1.01 to 1.33 (<5; Thompson et al., 2017; Lan and Wang, 2020a), indicating that the possible existence of multicollinearity issue was minimal.

Results of linear regression analysis are presented in Table 2. The model explained 11.2% (adjusted R^2^ = 10%) of the variance in depressive symptoms, *F* (17, 1,233) = 9.16, *p* < 0.001. To be specific, consistency (*b* = -1.77, *p* < 0.001) and social support (*b* = -0.97, *p* < 0.001) were negatively related to depressive symptoms, and fear of infection (*b* = 1.27, *p* < 0.001) was positively associated with depressive symptoms. However, the regression analysis did not exhibit significant and direct associations of infection risk, perseverance, and several confounding variables (i.e., age, gender, family SES, number, and time) with depressive symptoms^5^.

**TABLE 2:** Regression analysis predicting depressive symptoms from coronavirus disease 2019 (COVID-19) infection risk, grit, and social support.

**Variables**	***b***	***SE b***	**95% CI for *b***		***t***	***p***
Infection Risk (IR)	0.78	0.46	−0.11	1.68	1.72	0.09
Perseverance (PE)	−0.49	0.33	−1.13	0.15	−1.50	0.13
Consistency (CO)	−1.77	0.29	−2.33	−1.21	–6.17	<0.001
Social Support (SS)	−0.97	0.23	−1.42	−0.51	−4.17	<0.001
Age	0.11	0.14	−0.16	0.38	0.83	0.41
Male^*a*^	0.59	0.42	−0.23	1.41	1.40	0.16
Socioeconomic Status	−0.03	0.09	−0.20	0.15	−0.29	0.77
Fear of Infection	1.27	0.25	0.77	1.77	4.99	<0.001
Number	−0.27	0.17	−0.61	0.06	−1.62	0.11
Time	0.13	0.14	−0.16	0.41	0.89	0.37
IR × PE	−0.60	0.69	−1.95	0.75	−0.87	0.38
IR × CO	0.21	0.59	−0.95	1.37	0.35	0.73
IR × SS	−0.89	0.49	−1.84	0.07	−1.82	0.07
PE × SS	−0.01	0.23	−0.46	0.43	−0.05	0.96
CO × SS	0.31	0.23	−0.13	0.75	1.38	0.17
IR × PE × SS	1.19	0.51	0.19	2.18	2.33	0.02
IR × CO × SS	−0.54	0.48	−1.49	0.41	−1.11	0.27

*N = 1,251. ^*a*^Gender was regarded as a dummy variable, and we specified male as a reference group in this study. *b*, the unstandardized beta; *SE b*, the standard error for the unstandardized beta; CI, confidence interval. Number refers to the number of close family members present during quarantine, and time reflects how many hours per day young adults spent getting access to news and information related to COVID-19 in social media.*

With regard to the two-way interaction terms in the linear regression, no significant interaction was found. As regards the three-way interaction terms, the interaction among infection risk, perseverance, and social support was positively associated with depressive symptoms (*b* = 1.19, *p* < 0.001), whereas the interaction among infection risk, consistency, and social support was not significantly linked to depressive symptoms.

**FIGURE 1:**
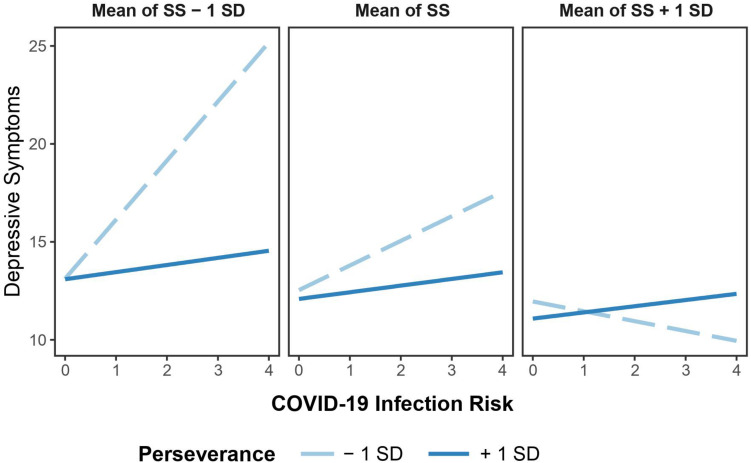
Interaction effect of coronavirus disease 2019 (COVID-19) infection risk, perseverance, and social support on depressive symptoms among Chinese young adults in quarantine. *N* = 1,251. SS, social support. Perseverance and social support were divided into two levels based on mean: low = *M*-1 *SD*, high = *M* + 1 *SD*.

A simple slope analysis exhibited that when reporting higher levels of social support, the association of COVID-19 infection risk with depressive symptoms was not significant at both higher levels of perseverance [*b* = 0.31, *SE* = 0.63, 95% CI = (-0.93, 1.56), *t* = 0.49, *p* = 0.62], and lower levels of perseverance [*b* = -0.57, *SE* = 1.03, 95% CI = (-2.59, 1.45), *t* = -0.55, *p* = 0.57]. In contrast, when reporting lower levels of social support, the association between COVID-19 infection risk and depressive symptoms was positively associated for young adults exhibiting lower levels of perseverance [*b* = 2.99, *SE* = 0.76, 95% CI = (1.50, 4.49), *t* = 3.92, *p* < 0.001] but not for those showing higher levels of perseverance [*b* = 0.39, *SE* = 1.14, 95% CI = (-1.84, 2.64), *t* = 0.34, *p* = 0.72; Figure 1]. From a descriptive point of view, in the context of lower social support, the levels of perseverance did not seem to be significantly associated with depressive symptoms for those who did not report infection risk; by contrast, for those who reported high infection risk, lower perseverance was associated with higher levels of depressive symptoms.

## Discussion

The COVID-19 pandemic seems to impact individuals’ lives in quarantine due to several intrinsic factors, including negative emotional impact. In this regard, it would be valuable to investigate the risk and protective factors associated with COVID-19 infection risk and depressive symptoms among young adults in quarantine. We examined the moderating role of two dimensions of grit and social support in this association using a large-scale sample of Chinese young adults in quarantine. The findings of this study revealed that COVID-19 infection risk was positively linked to depressive symptoms and that this association was moderated by perseverance and social support: for those reporting sufficient social support, the association between COVID-19 infection risk and depressive symptoms was not significant at both higher and lower levels of perseverance; in contrast, for those reporting a high infection risk for COVID-19 and limited social support resources, low perseverance was significantly associated with severe depressive symptoms.

The first goal of the present research was to examine the association between COVID-19 infection risk and depressive symptoms among young adults in quarantine. In line with the first hypothesis, results showed that COVID-19 infection risk was positively related to depressive symptoms. One possible explanation is that during quarantine, uncertainty about the future, disruptions to personal networks, and the experience of illness and bereavement among close family members and peers can bring salient stress to individuals and impair psychological functioning (Brooks et al., 2020; Ransing et al., 2020). Moreover, due to the absence of effective vaccines and treatments for COVID-19, concerns about physical safety may potentially increase individuals’ depression (Brooks et al., 2020). In addition, social isolation triggered by quarantine may make several stressors (e.g., job-seeking and pursuing higher education) more salient during young adulthood, which may further magnify their negative emotions (Arnett, 2000; Parola et al., 2020).

The second goal of this study was to investigate the moderating role of two dimensions of grit and social support in the relationship between COVID-19 infection risk and depressive symptoms. Results showed that when reporting higher levels of social support, the association between COVID-19 infection risk and depressive symptoms was not significant at both higher and lower levels of perseverance. Such a finding is in accordance with prior research highlighting the buffering role of social support on individuals’ depressive symptoms (Auerbach et al., 2011; Dumitrache et al., 2017). One possible explanation is that when young adults have suspected symptoms or have close contact with infected/suspected cases, available social relationships may provide an immediate coping response and may alleviate the impact of infection risk by providing an effective solution or by neutralizing the perceived concerns of COVID-19 (Grey et al., 2020); young adults with high social support are thus less reactive and sensitive to infection risk, resulting in lower levels of negative emotions. In contrast, in the context of lower levels of social support, high perseverance can buffer against depressive symptoms when young adults are exposed to increased infection risk. Such a finding is in accordance with prior research (Blalock et al., 2015; Lan and Radin, 2020), suggesting that individuals with high perseverance can push through hardships and lessen the attention they devote to infection risk by adjusting their focus to a long-term perspective.

However, the moderating effect of consistency in the aforementioned association was not significant. One possible explanation is ascribed to Chinese cultural values, in which group harmony and flexibility are highly emphasized (Chen, 2010). Young adults’ interests may be expected to adjust based on interdependent contexts and demands of significant others. Moreover, young adults in Chinese society are also likely to demonstrate analytical thinking, which underscores a high level of tolerance for conflicts and discrepancies (Talhelm et al., 2014). In this regard, consistency may not be a crucial factor for young Chinese adults’ psychological functioning and the emergence of depressive symptoms (Datu et al., 2016).

### Limitations

The current results should be interpreted within the following limitations. First, the present study is heavily constrained by a cross-sectional, correlational design. Thus, we cannot exclude inherent individual differences of study participants, and we are unable to infer causality with respect to the relationships observed between study variables. In light of this, future studies should use a multi-wave longitudinal design to estimate these understudied associations.

Second, COVID-19 infection risk is measured using a questionnaire with a dichotomous (yes/no) format that includes only four items. Although these items seem face-valid, the mean score of COVID-19 infection risk in this study is relatively low, and the correlation coefficient between COVID-19 infection risk and depressive symptoms is weak (*r* = 0.07, *p* < 0.05). One possible explanation could be that Chinese society highly values group well-being, cooperative skills, and behavioral constraints (Chen, 2010). In this regard, young adults may follow quarantine policies strictly, and thus their perception of infection risk is relatively low. Nevertheless, readers should also keep the limitation of this measure in mind when interpreting the current findings. Future studies should employ an ordinal response format and use more research items to obtain a more detailed picture of young adults’ infection risk with respect to COVID-19.

Third, we rely solely on a sample of Chinese college students from Hubei Province to address study associations, restricting the generalization of research findings to other populations and cultural backgrounds. In light of this, future studies should consider recruiting a nationally representative sample of young adults or conducting a cross-cultural investigation to replicate the current findings. Moreover, to gain a more comprehensive understanding of young adults’ emotional and behavioral adjustment during quarantine, it is highly recommended that future research includes other variables/constructs, such as externalizing problem behavior.

Finally, due to research constraints in quarantine, we rely heavily on self-report questionnaires to assess study variables. Although these measures are well-validated and widely used for young adults, we cannot entirely exclude the influence of common method bias and desirability bias on research findings, which may inflate the magnitude of study associations (Podsakoff et al., 2003). For example, it should be noted that the mean score of depressive symptoms in this study is relatively low. This may be because young adults tend to perceive themselves as invulnerable and often underestimate their risk of experiencing negative emotions (Millstein and Halpern-Felsher, 2002). Hence, future research should use diverse data collection methods, such as interviews and observations, to objectively determine the relationship between COVID-19 infection risk and depressive symptoms.

## Conclusion

The current study provides preliminary empirical evidence about risk and protective factors in the association between COVID-19 infection risk and depressive symptoms using a large-scale sample of young adults in quarantine. In the context of the COVID-19 pandemic, greater social support is essential to helping young adults in quarantine deal with negative emotions. In this regard, university-based counseling services should organize some online activities or meetings among college students, aiding them to build/maintain sufficient social networks during quarantine. Moreover, university-based counseling services should pay specific attention to those young adults with relatively insufficient social support resources and low levels of perseverance.

## Data Availability Statement

The datasets presented in this study can be found in online repositories. The names of the repository/repositories and accession number(s) can be found below: https://osf.io/8d6p9/.

## Ethics Statement

The studies involving human participants were reviewed and approved by the Institutional Review Board affiliated by the Jingchu University of Technology. The patients/participants provided their written informed consent to participate in this study.

## Author Contributions

JH conceived and drafted the manuscript. QY participated in data collection and interpretation of the results. XL performed the statistical analyses and critically revised the manuscript. All authors read and approved the final draft of the manuscript.

## Conflict of Interest

The authors declare that the research was conducted in the absence of any commercial or financial relationships that could be construed as a potential conflict of interest.

## Footnotes

1 We acknowledge that preregistration of empirical studies is valuable for improving the credibility and reproducibility of research findings (for elaboration, see van’t Veer and Giner-Sorolla, 2016) and has been encouraged by an increasing number of academic journals. However, it should be noted that the outbreak of COVID-19 in China and subsequent national lockdown and quarantine policies are regarded as suddenly happened public health events, and our research aim is to timely reflect how the impact of COVID-19 infection risk on young adults’ psychological distress and provide possible practical implications for university-based counseling services (if possible). As noted in the *Methods* section (i.e., data collection of this study was administered online in March 2020), we indeed have limited time to preregister our study aims and hypotheses. Also, due to the scarcity of prior knowledge on this issue, preregistration of the current study becomes more challenging, although it is not entirely impossible.

2 To reduce the transmission of COVID-19, the Chinese government launched various lockdown and quarantine policies (Xin et al., 2020). Hubei Province in China initially imposed the strictest quarantine measures (Kang et al., 2020). For example, most public transportation was suspended, and strict traffic control was then applied to minimize personal movements; individuals who contacted with persons with confirmed or suspected COVID-19 and those who traveled from or *via* Hubei had to isolate themselves for 14 days in designated facilities (e.g., governmental facilities and hotels) or home settings after arriving at their destinations; all shops, except for food supplies or pharmacies, shut down, and only one family member of each family was allowed to go out for buying necessities every 3 days; schools and universities were already in a period of winter holiday, but the opening days were uncertain and expected to be extended; volunteers and officials went door to door for health checks, distributing disinfection supplies and ensuring that all individuals abided by quarantine policies; most residence buildings were closed except for their inhabitants, and security guards were responsible for monitoring temperatures of all individuals going inside the residence (National Health Commission of China [NHCC], 2020a).

3 https://osf.io/8d6p9/

4 Adopting hierarchical linear regression may be appropriate to address our research objective. However, given that many predictors and interaction terms were entered into the linear regression, we employed multiple linear regression analysis for brevity. This is because the results obtained in the last step of hierarchical multiple regression are the same as those obtained in a single-step multiple linear regression (Mundfrom and Schults, 2002).

5 In the current study, we did not explore gender differences in study associations based on the following considerations. Conceptually, we aim to understand whether two dimensions of grit and social support moderate the association between COVID-19 infection risk and depressive symptoms. Therefore, unpacking gender differences in these study associations goes beyond our research aims. Statistically, based on our research objective, we already included three-way interaction terms (i.e., COVID-19 infection risk × perseverance/consistency × social support) in the linear regression model; in this regard, including the gender variable would yield a four-way interaction, which is usually difficult to interpret and is rarely meaningful. Perhaps more importantly, based on the results of bivariate correlations (Table 1), we did not find significant gender differences in depressive symptoms.
